# Succinylation profiles of brain injury after intracerebral hemorrhage

**DOI:** 10.1371/journal.pone.0259798

**Published:** 2021-11-15

**Authors:** Yuan-Hong Deng, Xin-Xiao Zhang, Chuan-Yuan Tao, Yan-Jing Liang, Jing Yuan, Su-Hao Yang, Yuan-Rui Yang, Xiao-Yi Xiong

**Affiliations:** 1 Acupuncture and Tuina School/Third Teaching Hospital, Chengdu University of Traditional Chinese Medicine, Chengdu, China; 2 Acupuncture & Chronobiology Key Laboratory of Sichuan Province, Chengdu, Sichuan Province, China; 3 Department of Neurosurgery, West China Hospital, Sichuan University, Chengdu, China; 4 Department of Geriatrics, The General Hospital of Western Theater Command, Chengdu, Sichuan, China; NIH, UNITED STATES

## Abstract

Protein posttranslational modifications (PTMs) regulate the biological processes of human diseases by genetic code expansion and cellular pathophysiology regulation; however, system-wide changes in PTM levels in the intracerebral hemorrhage (ICH) brain remain poorly understood. Succinylation refers to a major PTM during the regulation of multiple biological processes. In this study, according to the methods of quantitative succinyllysine proteomics based on high-resolution mass spectrometry, we investigated ICH-associated brain protein succinyllysine modifications and obtained 3,680 succinylated sites and quantified around 3,530 sites. Among them, 25 succinyllysine sites on 23 proteins were upregulated (hypersuccinylated), whereas 13 succinyllysine sites on 12 proteins were downregulated (hyposuccinylated) following ICH. The cell component enrichment analysis of these succinylproteins with significant changes showed that 58.3% of the hyposuccinylated proteins were observed in the mitochondria, while the hyper-succinylproteins located in mitochondria decreased in the percentage to about 35% in ICH brains with a concomitant increase in the percentage of cytoplasm to 30.4%. Further bioinformatic analysis showed that the succinylproteins were mostly mitochondria and synapse-related subcellular located and involved in many pathophysiological processes, like metabolism, synapse working, and ferroptosis. Moreover, the integrative analysis of our succinylproteomics data and previously published transcriptome data showed that the mRNAs matched by most differentially succinylated proteins were especially highly expressed in neurons, endothelial cells, and astrocytes. Our study uncovers some succinylation-affected processes and pathways in response to ICH brains and gives us novel insights into understanding pathophysiological processes of brain injury caused by ICH.

## Introduction

Intracerebral hemorrhage (ICH) is an extremely dangerous illness with an unfavorable prognosis and limited effective therapies [1]. Although the research progress of ICH-related molecular changes has been obtained at the genomic, transcriptomic, and proteomic levels [2–7], details on protein posttranslational modifications (PTMs) in the ICH brain tissues are still little known.

PTMs are among the most effective biological mechanisms, which can expand the genetic code, regulate cellular pathophysiology [8, 9], and have been proved to be participated in regulating the pathophysiological processes of diseases, like brain injury [10], cancer [11], and Alzheimer’s disease [12]. Protein succinylation, a novel PTM identified in 2011 [13], is important for its involvement in many biological processes, such as metabolic changes and inflammation, which occur by affecting enzymatic activity. For instance, the succinylation of enzymes involved in glucose and fatty acid metabolism could regulate cellular energy metabolism [14, 15]. Additionally, succinylation is critical for regulating innate immunity and inflammation [16]. These results strongly indicate that protein succinylation is being increasingly implicated in participating in the pathophysiological processes of human diseases. However, whether protein succinylation is also involved in the development of ICH remains largely unclear.

Here, an unbiased, site-specific succinylproteome profiling based on high-resolution mass spectrometry was performed to characterize protein succinylation in mouse ICH and control (sham) brains. This study gives us an unprecedented view of ICH-associated brain protein succinylation modifications. The further bioinformatic analysis illustrated that succinylproteins were characterized by primarily locating in mitochondria and synapse-related space and participating in many biological processes. Moreover, the combined analysis of previously published transcriptome data of neural cells with our succinylproteome data revealed that these significantly altered succinylproteins were mainly distributed in neurons, endothelial cells, and astrocytes. Our study uncovers some novel therapeutic targets of the succinylation-involved processes and pathways in response to brain injury after ICH and gives us novel insights into understanding biological processes of ICH-induced brain damage.

## Results

### Succinylproteome profiling analysis of ICH and control brains

Label-free LC-MS/MS was performed on perihematomal brain tissues (ICH) and control (sham) brain samples three days post-ICH. The FDR was set as 1% in accuracy identification on the spectrum, peptide, and protein levels (S1A Fig). Most peptides had a length of 7–20 amino acids (S1B Fig), and the corresponding first-order mass error in most spectra was less than 10 ppm (S1C Fig), suggesting that the succinylproteomics data recognized by mass spectrometry satisfied the relevant quality control demands. Principal component analysis (PCA) was performed to evaluate the repeatability of the succinylproteome data, and the results showed better biological repeatability in the samples (S1D Fig). The results of the sequence motif analysis of the succinylpeptides revealed five conserved sequences around lysine succinyllysine sites were detected in the succinylproteome (S1E and S1F Fig). Additionally, Ala, Gly, Ile, and Val occurred frequently around the succinyllysine residues (S1E Fig).

**Fig 1:**
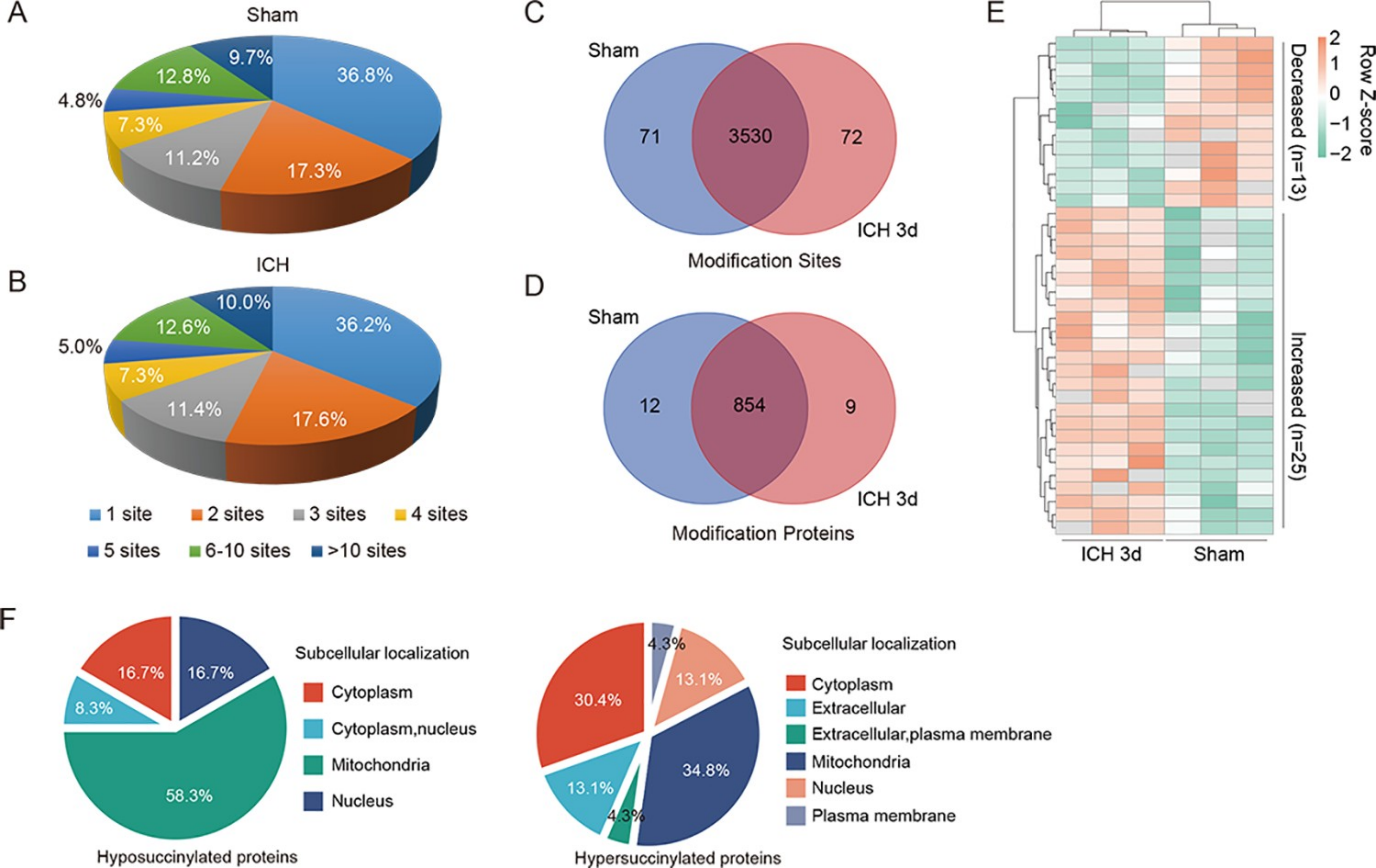
Quantitative succinylproteomics analysis of ICH and control brains. (A-B) Pie charts showing the proportions of singly and multiple succinylated proteins with the indicated number of succinylated sites per protein in control (sham) (A) and ICH (B) brains in vivo. (C-D) Venn diagram comparing succinylated sites (C) and proteins (D) between ICH and control brains. (E) The heat map shows the differential succinylated sites in ICH and control brains. (F) Subcellular localization of hyposuccinylated (right) or hypersuccinylated (left) proteins in ICH and control brains.

We identified around 3,680 succinylated sites in 895 modified proteins and quantified approximately 3,500 sites (Fig 1A and S1 Table). Among these modified proteins in the Control tissues, approximately 37% of the succinylated proteins had one succinyllysine site, 17% of them had two succinyllysine sites, 46% had more than three succinyllysine sites, and approximately 10% had more than 10 succinyllysine sites (Fig 1A). The percentage of succinylproteins in the ICH brains was comparable to that in the control brains (Fig 1A and 1B). The Venn diagram showed that over 96% of the succinyllysine sites (3530/3673, Fig 1C) and succinylproteins (854/875, Fig 1D) were similar between the ICH and control brains. Among them, 72 succinyllysine sites on 9 succinylated proteins were found only in the ICH brains (Fig 1C and 1D). Conversely, 71 succinyllysine sites on 12 succinylated proteins were found only in the control brains (Fig 1C and 1D). Furthermore, in response to ICH-induced brain injury, 25 succinyllysine sites on 23 succinylated proteins were upregulated (hypersuccinylated), whereas 13 succinyllysine sites on 12 succinylated proteins were downregulated (hyposuccinylated), as shown by the heat map (Fold Change > 1.5 and *P* < 0.05; Fig 1E; the mass spectrum peak map of some of the representative modified proteins are presented in S2 Fig). Additionally, subcellular localization analysis of these succinylated proteins with significant changes was performed, and the results showed that 58.3% of the hyposuccinylated proteins could be found in the mitochondria (Fig 1F). Interestingly, the ratio of hyper-succinylprotein in mitochondria localization reduced to approximately 35%, inversely increased to 30.4% in the cytoplasm in ICH brains (Fig 1F). These results indicated that the alterations in succinylation may primarily influence the biofunctions of the mitochondrial and cytoplasmic proteins.

### Annotation of differentially succinylated proteins in the brains of intracerebral hemorrhage

**Fig 2:**
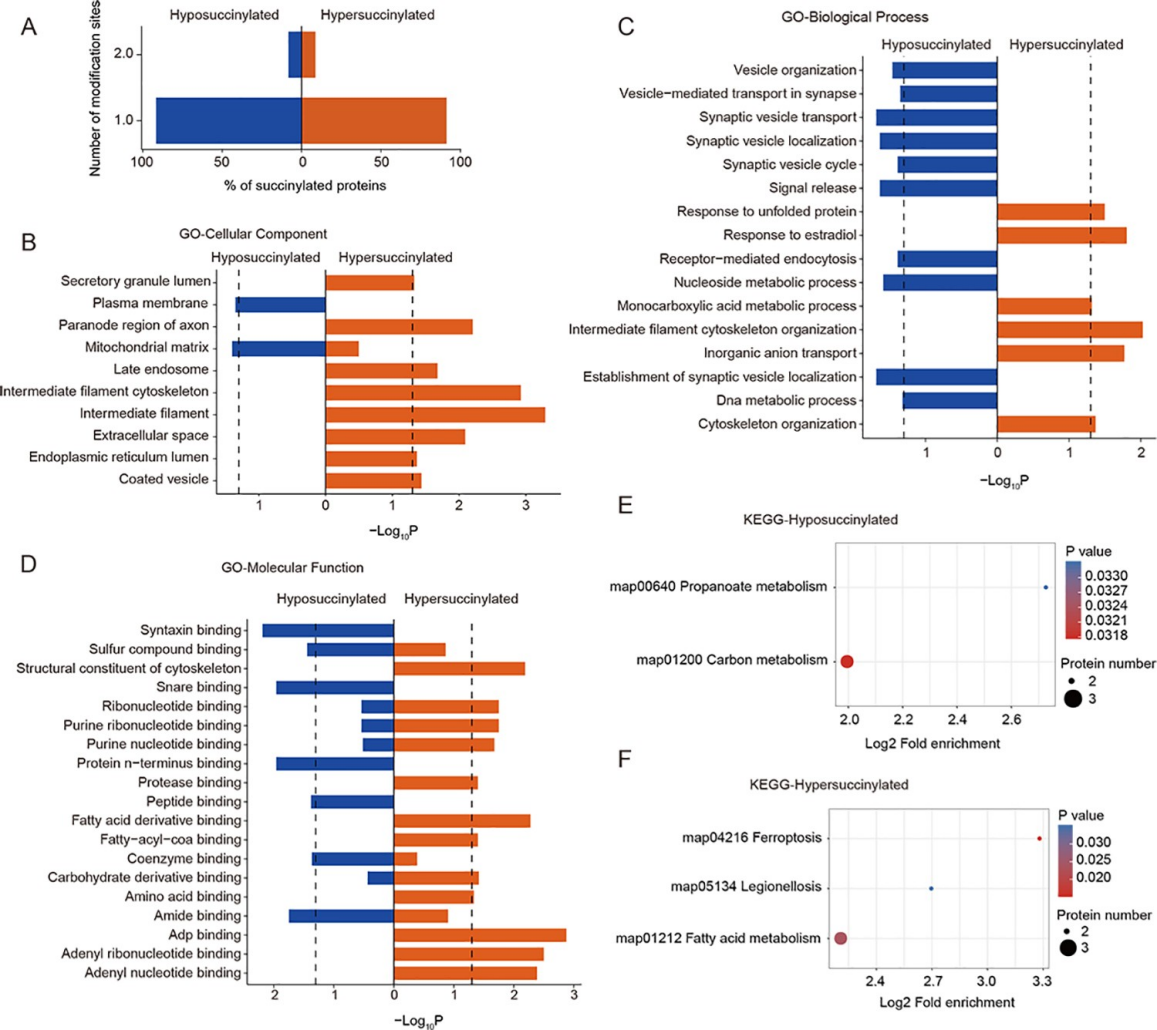
Analysis and annotation of differentially succinylated proteins in ICH brains. (A) Distribution of ICH-associated hyposuccinylated or hypersuccinylated proteins carrying the indicated number of succinylated sites per protein in vivo. The X-axis represents the percentage of hypo-and hyper-succinylated proteins (FC > 1.5 and *P* < 0.05) carrying 1 or 2 modification sites. The Y-axis represents the number of modification sites of hypo-and hyper-succinylated proteins (FC > 1.5 and *P* < 0.05). (B to D) GO analysis at the level of cellular component, biological process, and molecular function enriched in hyposuccinylated or hypersuccinylated proteins after ICH. (E and F) KEGG pathway analysis of hyposuccinylated or hypersuccinylated proteins after ICH. Significant enrichment is shown by Benjamini-Hochberg FDR-corrected *P* < 0.05 (outside the dashed line).

We found that the ICH-induced hyposuccinylation was comparable to hypersuccinylation as they both carried one or two succinyllysine sites in every succinylated protein in vivo (FC > 1.5 and *P* < 0.05; Fig 2A). Cellular component enrichment analysis of Gene Ontology (GO) revealed that both hyposuccinylated and hypersuccinylated proteins were located in the mitochondrial matrix, especially the hyposuccinylated proteins (Fig 2B), which is consistent with the results of the subcellular distribution data. Only ICH-associated hypersuccinylation had a significant correlation with the secretory granule lumen, paranode region of the axon, late endosome, intermediate filament cytoskeleton, intermediate filament, extracellular space, endoplasmic reticulum lumen, and coated vesicle (Fig 2B), whereas only the ICH-associated hyposuccinylation gained enrichment on plasma membrane (Fig 2B), suggesting that the hypersuccinylation-involved biofunctions may be more wide than those associated with hyposuccinylation in response to ICH-induced brain injury. However, in contrast to the results of the GO localization enrichment, the number of biological processes associated with ICH-associated hyposuccinylation was greater than that associated with hypersuccinylation (Fig 2C). For example, hyposuccinylated proteins in ICH brains were selectively enriched for vesicle organization, vesicle-mediated transport in synapse, the transport, localization, and cycle of synaptic vesicle, signal release, receptor-mediated endocytosis, nucleoside metabolic process, establishment of synaptic vesicle localization, and DNA metabolic process (Fig 2C). However, only hypersuccinylated proteins of the ICH brains could be alternatively enriched in GO terms associated with response to unfolded protein, response to estradiol, monocarboxylic acid metabolic process, intermediate filament cytoskeleton organization, inorganic anion transport, and cytoskeleton organization (Fig 2C). GO molecular function enrichment analysis showed significant enrichment of hyposuccinylation and hypersuccinylation datasets in sulfur compound binding, ribonucleotide binding, purine ribonucleotide binding, purine nucleotide binding, coenzyme binding, carbohydrate derivative binding, and amide binding (Fig 2D). However, only ICH-related hypersuccinylation was closely related to the structural constituent of the cytoskeleton, protease binding, fatty acid derivative binding, fatty-acyl-CoA binding, amino acid binding, ADP binding, adenyl ribonucleotide binding, and adenyl nucleotide binding (Fig 2D), whereas ICH-associated hyposuccinylations were only enriched for syntaxin binding, snare binding, protein N-terminus binding, and peptide binding (Fig 2D). Additionally, KEGG analysis of succinylated proteins with significant alteration showed that ICH-associated hyposuccinylation was enriched for propanoate metabolism and carbon metabolism (Fig 2E), while hypersuccinylation was significantly related to ferroptosis, fatty acid metabolism, etc. (Fig 2F). These results suggested that protein succinylation may be involved in many pathophysiological processes and pathways in response to ICH-induced brain injury.

**Fig 3:**
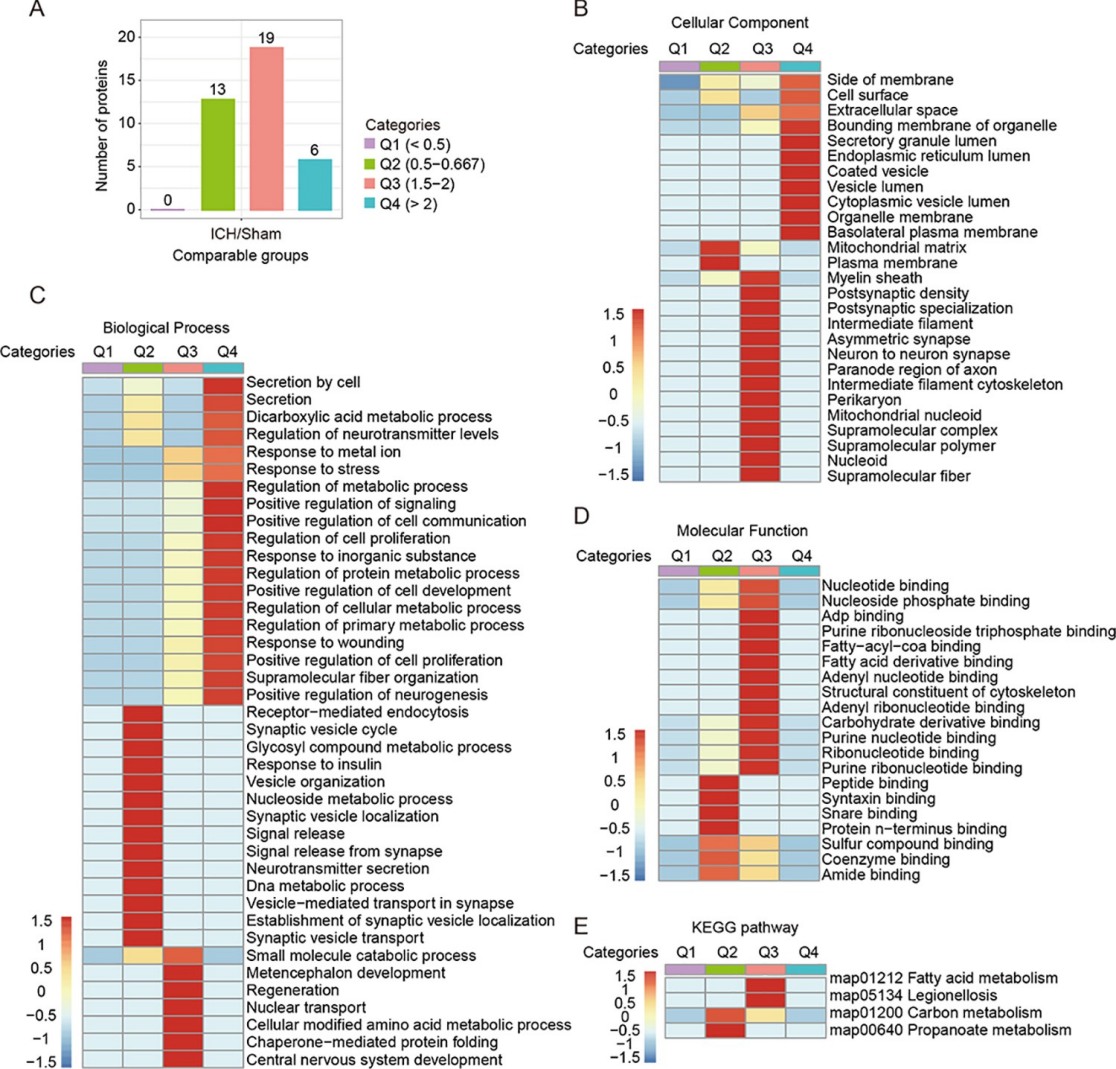
Four clusters of differentially expressed proteins were analyzed and annotated. (A) The number of differentially succinylated sites (Fisher’s exact test, *P* < 0.05) in each cluster after ICH. (B-D) GO analysis at the level of cellular component, biological process, and molecular function enriched in each cluster after ICH. (E) KEGG pathway analysis of each cluster after ICH.

Next, we classified these significantly altered succinylated proteins in the ICH brains into four categories according to their ratio of distribution (Fig 3A and S2 Table). The heatmap showed that the GO cellular component and biological process enrichment of these succinylation datasets were significantly linked to categories Q2-Q4 (Fig 3B and 3C), while GO molecular function enrichment and KEGG pathway analysis were only closely related to categories Q2 and Q3 (Fig 3D and 3E). For example, GO cellular component enrichment analysis showed that the Q4 category was related to membranes, including cell surface, endoplasmic reticulum, vesicle, organelle, and plasma; the Q2 category was related to the mitochondrial matrix and plasma membrane, while the Q3 category was linked to neuronal functions, such as axon, synapse, filament, etc. (Fig 3B). These results indicated that the altered levels of protein succinylation may have functional preferences for some proteins and play important roles in regulating the brain injury caused by ICH.

### Integrative analysis of transcriptome and succinylproteome analysis in brains of intracerebral hemorrhage

**Fig 4:**
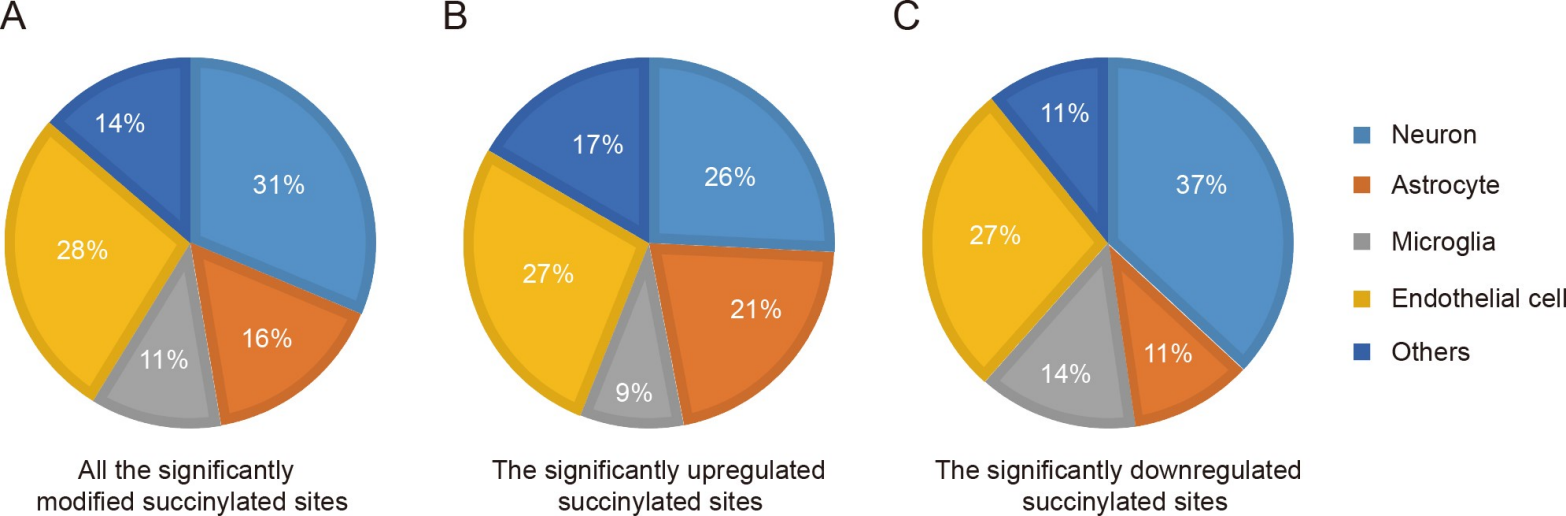
Combined analysis of succinylproteomics and transcriptomics data. (A-C) Pie charts showing the major cellular distribution of differentially succinylated sites (A), significantly upregulated (B), and downregulated succinylated sites (C) after ICH. Matching the significantly altered proteins (Student’s t-test, *P* < 0.05) with the relatively highly expressed genes of neural cells (the gene expression of the four main neural cell types was higher than that in the other cell types) from the published transcriptome data [17].

Given that four main neural cell types (i.e., neurons, astrocytes, microglia, and endothelial cells) were most studied in stroke, integrative analysis on the transcriptome data [17] of neural cells from mouse cerebral cortex RNA-seq with succinylation data to uncover cell type information regarding dramatically changed protein succinylation in response to ICH. Before the conjoint analysis, we first defined the relatively high expression of genes in neural cells as higher expression of genes in one type of neural cells than that in the other three types of cells. Through the match between the altered succinylproteomics data with the relatively high expression of genes in neural cells showed that several mRNAs matched by succinylproteins were relatively highly expressed in neurons, endothelial cells, as well as astrocytes in response to brain injury caused by ICH (Fig 4A). Moreover, the mRNAs matched by the 27% ICH-related upregulated succinylated proteins were found to be 27% from endothelial cells, 26% from neurons, 21% from astrocytes, and 9% from microglia (Fig 4B). The ratio of ICH-related downregulated succinylated proteins increased to 37% within neurons and 14% within microglia, accompanied by the reduction of percentage in astrocytes and microglia but with no significant changes in endothelial cells (Fig 4C).

## Discussion

This study provides a system-view of the succinylproteome profiles of ICH brains. Based on high-resolution LC-MS/MS and affinity with anti-succinyllysine agarose beads, we quantified 3,602 succinylated sites in 863 proteins in mouse ICH brains. This research illustrates to us that the succinylated proteins significantly changed, were primarily localized in mitochondria and cytoplasm, and were involved in many metabolic processes, neuronal functions, and ferroptosis. Furthermore, an integrative analysis of published transcriptome data and succinylproteomics data showed that neurons, endothelial cells, and astrocytes may be the major cells to regulate their biological functions in response to protein succinylation after ICH. This research gives us a landscape view of the mouse ICH brain succinylproteome, which may be a useful resource to further investigate the pathophysiological processes of brain damage caused by ICH.

Our quantitative succinylproteomic analysis revealed ICH-associated changes in the brain succinylproteome. We found that 38 succinyllysine sites from 35 succinylated proteins were significantly altered in ICH brains, including 25 and 13 succinyllysine sites with increased and decreased occupancy, respectively. Additionally, some succinyllysine sites on succinylated proteins could only be found in the ICH or control brains. Such site-specific changes in succinylation occupancy are possibly caused by alterations in the conformation of disease-related proteins, which influence the pathophysiological processes of brain injury via the succinylation machinery after ICH.

The study found 35 differentially succinylated proteins recognized from ICH brains, where 23 proteins were hypersuccinylated and 12 proteins were hyposuccinylated. The results of the GO and KEGG analysis for such differentially succinylated proteins revealed that protein hypersuccinylation and hyposuccinylation strongly influence biological processes associated with disease progression following ICH. Hyposuccinylation, but not hypersuccinylation, was preferentially associated with proteins localized to the mitochondrial matrix and plasma membrane, while only hypersuccinylation was preferentially related to proteins localized to the endosome, ER, vesicle, cytoskeleton, etc. Additionally, an integrative analysis with previously published transcriptome data showed that protein succinylation in brain tissues was mostly found in neurons, endothelial cells, and astrocytes following ICH, suggesting that succinylation may influence their functions associated with the pathophysiological processes in brain damage caused by ICH. Furthermore, KEGG pathway analysis on hypersuccinylated proteins demonstrated the involvement of succinylated proteins in ferroptosis, which is a contributor to post-ICH brain injuries [18–22], and fatty acid metabolism, which has been shown to be influenced by protein succinylation [14, 23, 24], indicating that protein succinylation-mediated fatty acid metabolism may occur in ICH-induced brain injury, although this was largely unknown to date. Therefore, our findings, along with recent studies, suggested that protein succinylation may participate in regulating the pathophysiological processes and pathways of ICH-induced brain damage. However, given that the results of this study were only based on quantitative succinylproteome, further researches still need to be done for validation of the molecular roles and mechanisms of the changes regarding ICH-associated protein succinylation.

## Conclusions

This is the first study to investigate the alterations of protein succinylation profiles in response to ICH-induced brain injury. Our study showed that changes in the succinylproteome may play an important role in ICH-induced brain damage. The identification of differential protein succinylation in ICH brains presents a new perspective on ICH pathogenesis at the molecular and system levels and provides potential therapeutic targets for ICH that were previously unknown.

## Materials and methods

### Animals

Experimental animals, i.e., male C57BL/6 mice (eight weeks old, 20–24 g), were purchased from Byrness Weil Biotech. Ltd. (Chengdu, China). The mice were bred under controlled temperatures in a specific pathogen-free (SPF) atmosphere, during a 12-hour light/dark cycle, with freely available food and water. The experimental procedures were approved by the Animal Ethics Committee at Chengdu University of Traditional Chinese Medicine.

### Collagenase-induced mouse model of ICH

The ICH model of mice was established according to previously described methods [25]. Briefly, mice were anesthetized with 3% isoflurane for induction and placed in a stereotaxic instrument (RWD Life Science Co., Shenzhen, China). Then, 1.5% isoflurane was used for maintenance to minimize the suffering of the mice during surgery. Approximately 0.5 μL of 0.075 IU type VII collagenase (Sigma Aldrich) was injected at a rate of 0.0625 μL/min into the left striatum (anterior: 0.8 mm, lateral of bregma: 2 mm, depth: 3.5 mm) using a syringe pump (Hamilton, Bonaduz, AG). In contrast, 0.5 μL of saline was injected into the control (sham) group. During the surgery, the body temperature of the animals was maintained at 37°C. After surgery, the mice were monitored at least once a day, and no accidental deaths were observed. Three days after ICH, the mice were euthanized by cervical dislocation under deep isoflurane anesthesia. Then, perihematomal and contralateral brain tissues were obtained to perform the succinylproteome analysis.

### Brain sample preparation for LC-MS/MS

Brain tissues were milled within liquid nitrogen, suspended using lysis buffer (a cocktail of 8 M urea, 10 mM dithiothreitol (DTT), 50 mM nicotinamide (NAM), 3 μM trichostatin A (TSA), 0.1% protease inhibitor), and sonicated three times on ice. The supernatant was acquired by centrifugation (12,000 g, 4°C, 10 min). Protein concentration was determined using the BCA kit by following the specifications of the manufacturer. Protein digestion was performed as mentioned above [26].

We obtained the peptide mixes according to previously published methods [27]. To collect the succinylpeptides, tryptic peptides were dissolved with NETN buffer, and the affinity enrichment of the modified peptide was performed as previously described [28]. In brief, total peptides were incubated overnight with anti-succinyllysine agarose beads (PTM Biolabs, China) at 4°C, with mild shaking. The bound peptides were eluted from the beads using 0.1% trifluoroacetic acid and dried by vacuum centrifugation. To prepare the peptides for LC-MS/MS analysis, they were dissolved in 0.1% formic acid (FA) and desalted using C18 ZipTips (Millipore).

### LC-MS/MS analysis

LC-MS/MS analysis was conducted using the above-mentioned procedures [29]. In brief, succinylpeptides were dissolved with solution A (0.1% formic acid and 2% acetonitrile), followed by separation via EASY-nLC 1200 UPLC system using reverse-phase pre-column (Thermo Fisher Scientific). The flow rate of solution B reached 500 nL/min, and the corresponding gradient reached 9%-25% (0.1% formic acid in 90% ACN) for 36 min, 25%-35% for 18 min, 35%-80% for 3 min, and was maintained at 80% for 3 min. The separated peptides came under the NSI source and were analyzed with the Q ExactiveTM HF-X (Thermo Fisher Scientific), at 2.2 kV electrospray voltage, 120,000 MS scan resolution within scan scope between 350 and 1,600 *m/z*, and 28% NCE HCD fragmentation. These fragments could be observed from Orbitrap when the resolution was 15,000. The fixed first mass was determined to be 100 *m/z*. The automatic gain control (AGC) target was 1E5, the intensity threshold was 5E4, and the maximum injection time was 50 ms. Three biological replicates were performed.

### Database search

MaxQuant (v1.6.15.0) was used for retrieving the raw MS/MS data of the experiment. The database search was conducted based on parameters as follows: cleavage enzyme: Trypsin/P; maximum missing cleavages: 4; mass tolerance for precursor ions during the first/main search: 20/4.5 ppm; mass tolerance for fragments: 0.02 Da. A carbamidomethyl on Cys was considered to be the fixed modification, while succinylation and oxidation on Met were considered to be variable modifications. The corresponding false discovery rate (FDR) cutoff was adjusted to < 1%, the score for modified peptides was set at > 40, and a localization probability was > 0.75.

### Bioinformatic analysis

The bioinformatic analysis was conducted using the Perl package in the R language. Statistical analysis of the succinylproteome was conducted according to the logarithmic intensity of the values of each quantified protein obtained from the experiment. Motif characteristic analysis of the succinyllysine sites was performed using the MoMo analysis tool according to the Motif-X algorithm. Three biological repeats were performed, and a *P*-value of 0.05 was set as the cutoff. In Student’s t-test, a *P*-value below 0.05 was used to determine the statistical significance. The relative quantitative values at every modification site between ICH and control (sham) brains were compared based on the differentially succinyllysine sites. Kyoto Encyclopedia of Genes and Genomes (KEGG) was used for pathway annotation. Gene Ontology (GO) annotation was performed in different categories, such as cellular component, biological process, and molecular function. For each category, the significant cutoff, determined by the corrected *P*-value, was set at 0.05.

### Conjoint analysis of succinylproteome data and transcriptome data

Conjoint analysis was conducted using succinyl proteome data and formerly disclosed transcriptome data [17]. FPKM (fragments per kilobase of transcript per million mapped reads) was selected as the mRNA quantitative criterion. Based on the transcriptome data, we focused on the four most studied neural cells (neurons, astrocytes, microglia, and endothelial cells) in stroke. Neural cell genes with relatively high expressions were observed from the analysis for the higher expression of such genes in one of the four most surveyed types of neural cells, among the other three cellular types. Next, the differentially succinyllysine proteins were matched with transcriptome data to determine the cellular information of the succinylproteins.

## Supporting information

S1 Fig Analysis of succinylproteomic data in ICH and control brains. (A) Overview of the identification of protein succinylation. (B and C) Data quality control of peptide length distribution (B) and peptide mass tolerance distribution (C). (D) PCA showing the degree of dispersion of the succinylproteome in ICH and control (sham) brains. (E) Amino acid sequence properties of succinylated sites. The heat map shows significant position-specific underrepresentation or overrepresentation of amino acids flanking the succinylated sites. (F) Succinylation motifs and conservation of succinylated sites. The height of each letter corresponds to the frequency of that amino acid residue at that position. The central K refers to the succinylated Lys residue.
(TIF)

S2 Fig MS spectrum of the representative succinylated sites. MS/MS spectra of P01837_K100su, P03995_K108su, P47791_K448su, P16546_K1311su, Q922B1_K146su, Q921I1_K26su, Q99KE1_K496su, and Q9WUM5_K54su.
(TIF)

S1 Table The information of identified succinyllysine sites in this study. (XLSX)

S2 Table The category information of significantly altered succinylated proteins in the ICH brains according to their ratio of distribution. (XLSX)
