# Supplementary figures and images for: Succinylation profiles of brain injury after intracerebral hemorrhage
Source: PLoS One. 2021 Nov 15;16(11):e0259798. doi: 10.1371/journal.pone.0259798 (PMC8592435; doi:10.1371/journal.pone.0259798)

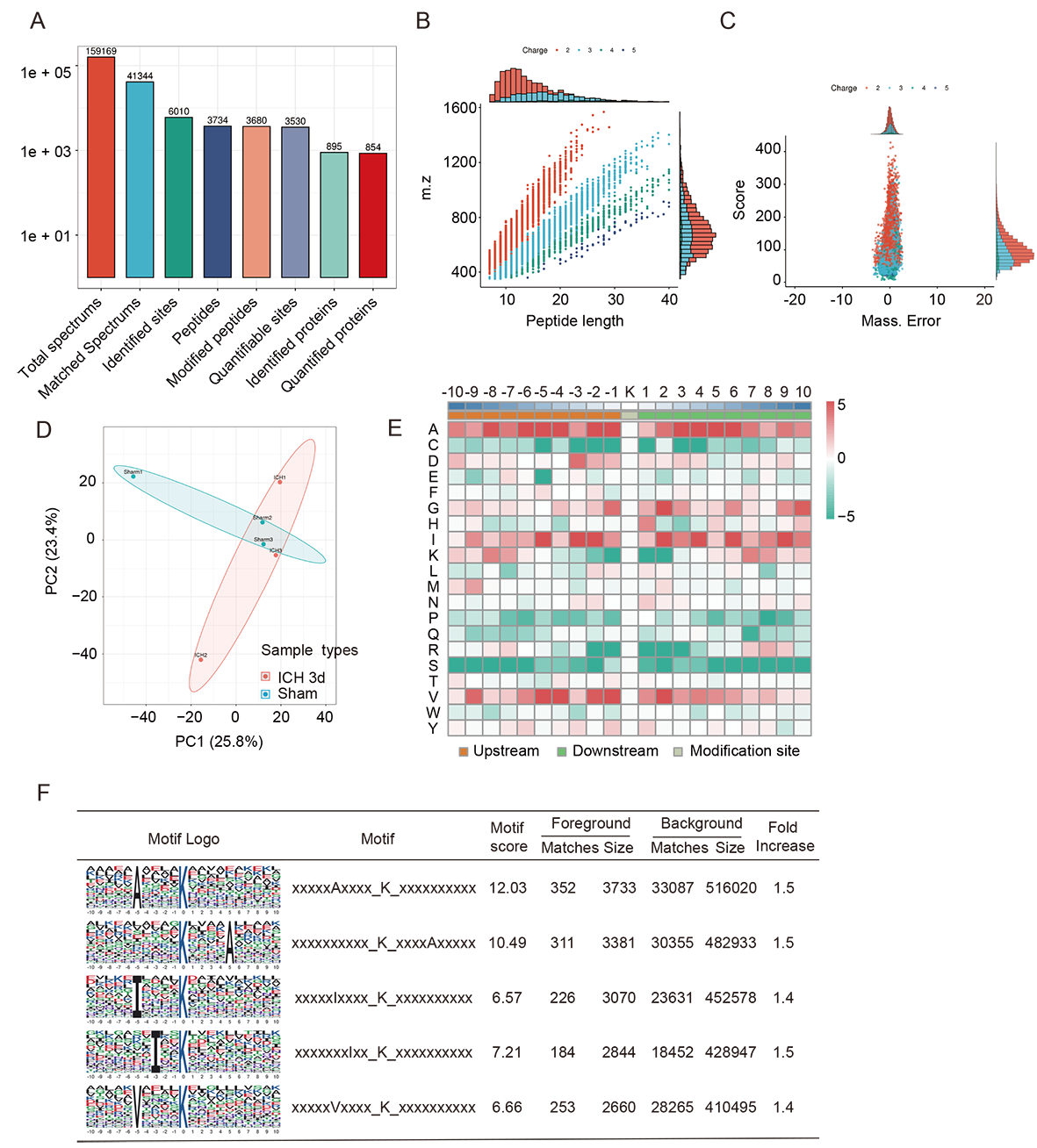

Supplement: S1 Fig — (A) Overview of the identification of protein succinylation. (B and C) Data quality control of peptide length distribution (B) and peptide mass tolerance distribution (C). (D) PCA showing the degree of dispersion of the succinylproteome in ICH and control (sham) brains. (E) Amino acid sequence properties of succinylated sites. The heat map shows significant position-specific underrepresentation or overrepresentation of amino acids flanking the succinylated sites. (F) Succinylation motifs and conservation of succinylated sites. The height of each letter corresponds to the frequency of that amino acid residue at that position. The central K refers to the succinylated Lys residue. (TIF) [file pone.0259798.s001.tif]

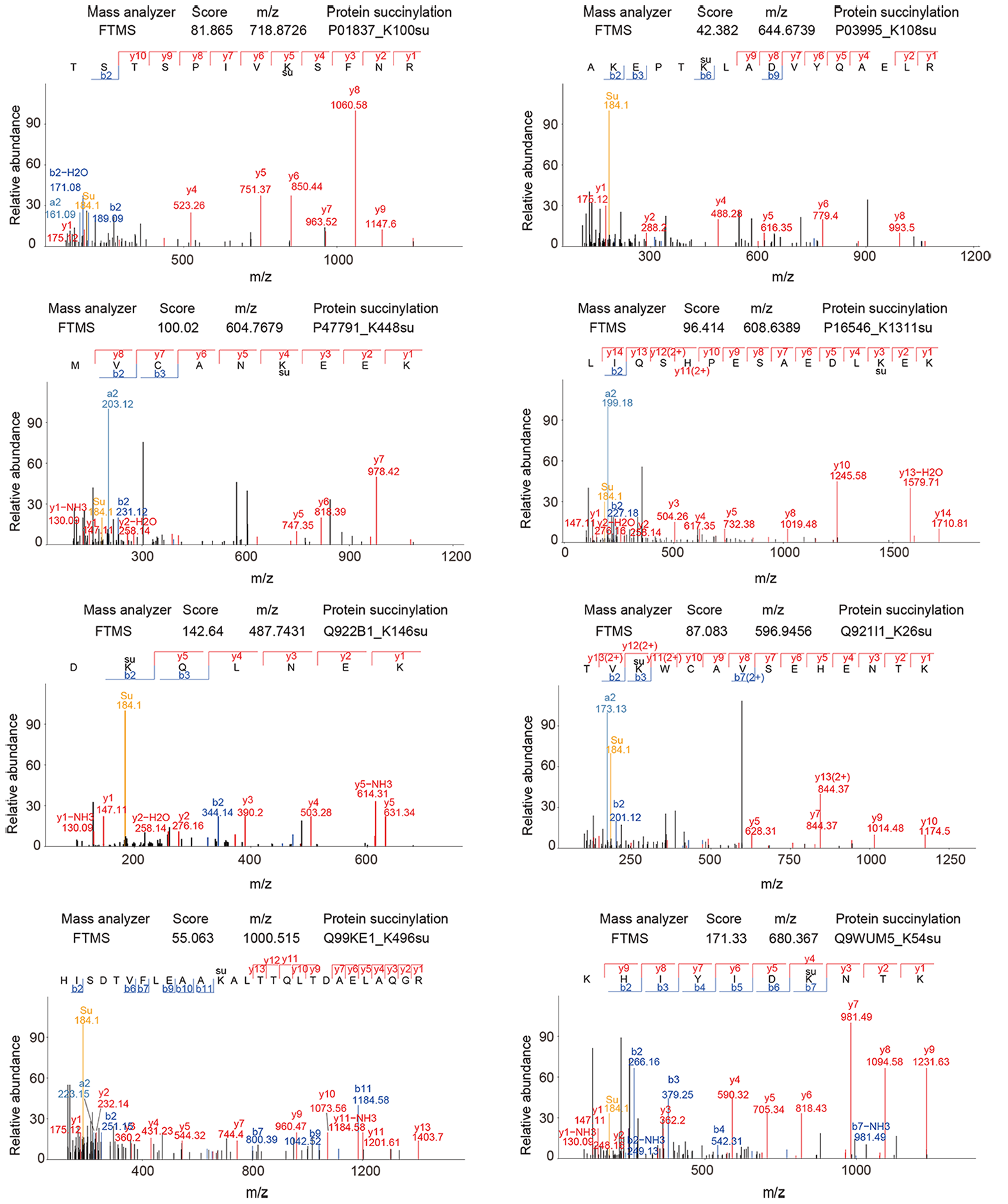

Supplement: S2 Fig — MS/MS spectra of P01837_K100su, P03995_K108su, P47791_K448su, P16546_K1311su, Q922B1_K146su, Q921I1_K26su, Q99KE1_K496su, and Q9WUM5_K54su. (TIF) [file pone.0259798.s002.tif]
